# Supplementary material for: Enhanced Resistance of atnigr1 against Pseudomonas syringae pv. tomato Suggests Negative Regulation of Plant Basal Defense and Systemic Acquired Resistance by AtNIGR1 Encoding NAD(P)-Binding Rossmann-Fold in Arabidopsis thaliana
Source: Antioxidants (Basel). 2023 Apr 24;12(5):989. doi: 10.3390/antiox12050989 (PMC10215446; doi:10.3390/antiox12050989)
Supplement: Supplementary file 1 [file antioxidants-12-00989-s001.zip › antioxidants-2248427-supplementary.pdf]

Supplementary Table S1. List of the primers used in this study

S.NO	Name	Forward primer (5′-3′)	Reverse primer (5′-3′)
1	*AtNIGR1* (At1g66130) (Genomic DNA)	CCGGAGCAAGTATTGGAAAG	CAACAAGTTTCACAGATGAA
2	*AtNIGR1* (At1g66130) (CDS)	GTTGGCACTATCTTGTCC	CTTTTGAGCCTGAGATAGC
3	*AtPR1* (AT2G14610)	GTGCAATGGAGTTTGTGGTC	TCACATAATTCCCACGAGGA
4	*AtPR2* (AT3G57260)	CAGATTCCGGTACATCAACG	AGTGGTGGTGTCAGTGGCTA
5	*AtAZI* (AT4G12470)	GCAAGCCAAGTCCTAAACCA	GTCGACGTCAACCAAACCTT
6	*AtG3Pdh* (AT2G41540)	CGTCTTTTGGGGAAATCAGA	GACATTGTCAATCGGCACAC
7	*AtActin2* (AT3G18780)	GCTGGACGTGACCTTACTGA	CCATCTCCTGCTCGTAGTCA
8	Salk LB 1.3 (T-DNA LB)	ATTTTGCCGATTTCGGAAC	
9	35SF	CTATCCTTCGCAAGACCCTTC	

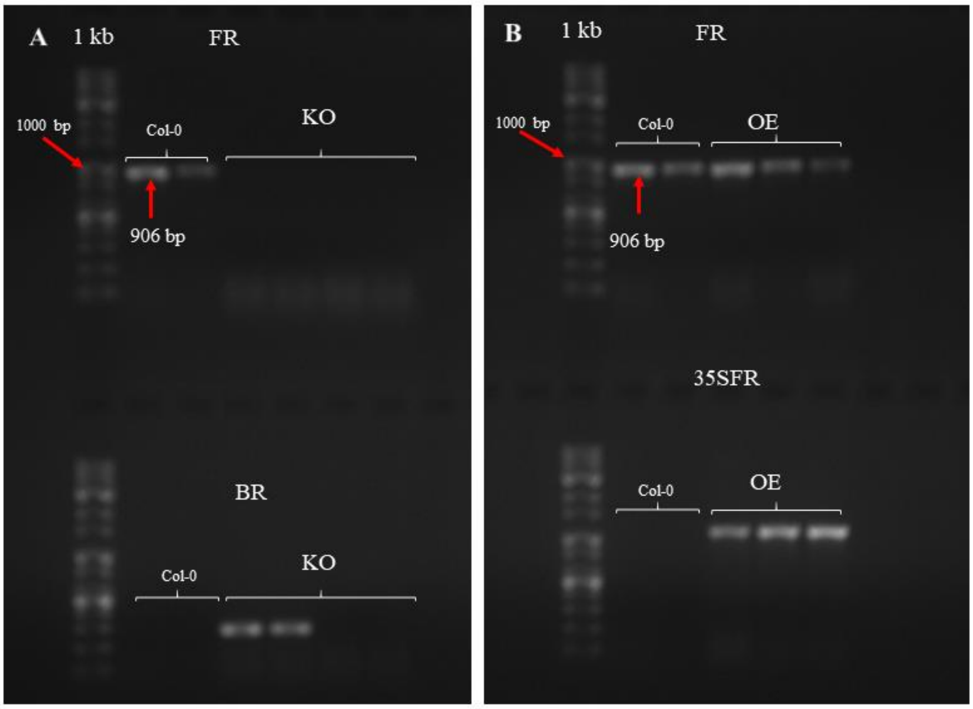

Supplementary Figure S1. Genotyping of *AtNIGR1* KO and OE plants. (**A**) Genotyping of *AtNIGR1* KO with forward and reverse primers and border and gene-specific reverse primers. (**B**) Genotyping of *AtNIGR1* OE with forward and reverse primers and 35S forward and gene-specific reverse primers.

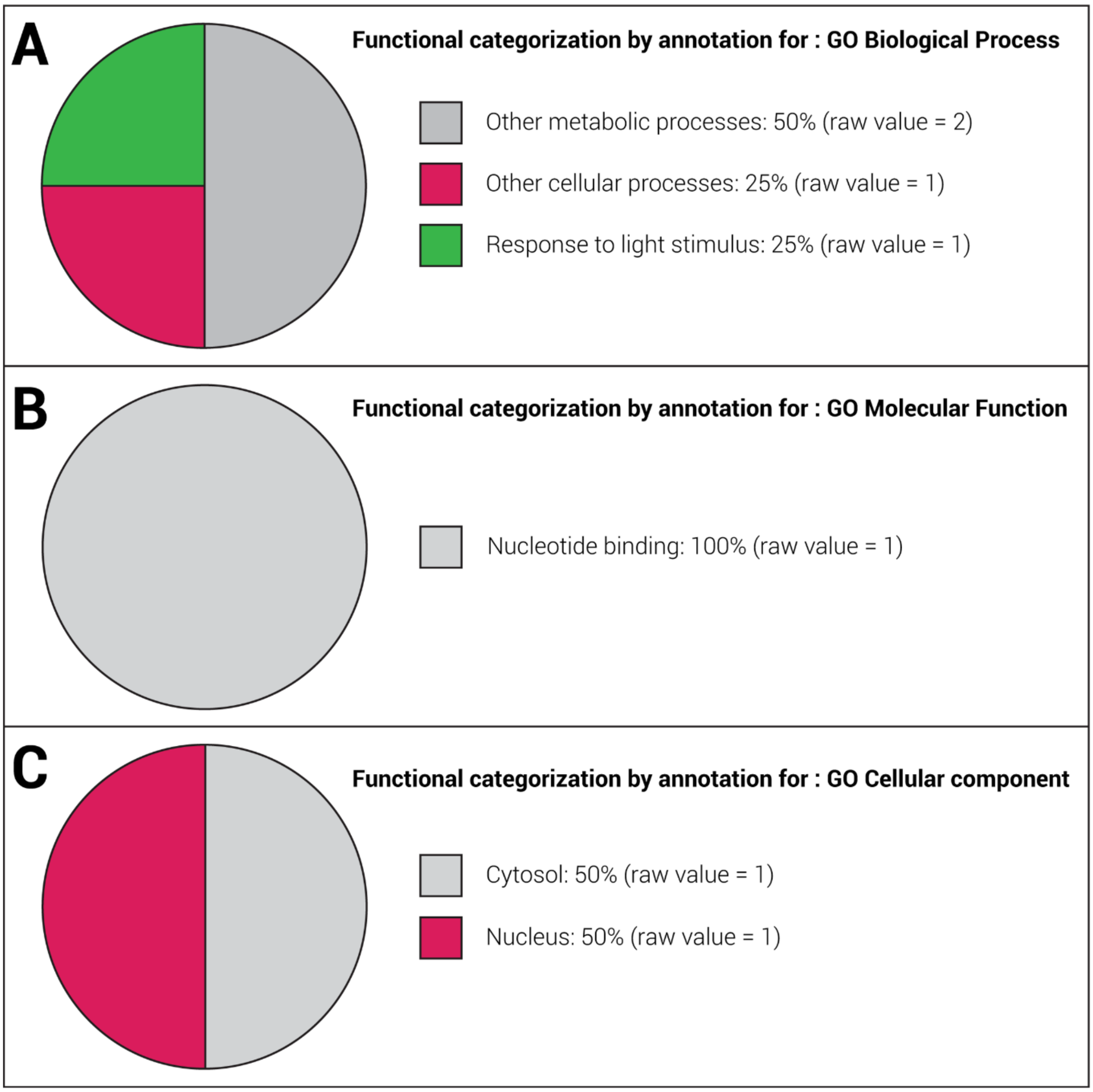

GO Annotations Locus	Gene Model(s)	GO (GO ID)	Code	GO Slim	Reference	Made by: date last modified
AT1G66130	AT1G66130	Response to blue light (GO:0009637)	IEA	Response to light stimulus	Publication: 501796011|PMID:34562334	NA10/15/202100:00:00
	AT1G66130	Pigment biosynthetic process (GO:0046148)	IEA	Other metabolic processes	Publication: 501796011|PMID:34562334	NA10/15/202100:00:00
	AT1G66130	Cytosol (GO:0005829)	HDA	Cytosol	Publication: 501761766|PMID:25293756	NA04/04/201900:00:00
	AT1G66130	Nucleus (GO:0005634)	HDA	Nucleus	Publication: 501776792|PMID:28887381	NA04/04/201900:00:00
	AT1G66130	Chlorophyll metabolic process (GO:0015994)	IEA	Other cellular processes<br>Other metabolic processes	Publication: 501796011|PMID:34562334	NA10/15/202100:00:00
	AT1G66130	Nucleotide binding (GO:0000166)	IEA	Nucleotide binding	AnalysisReference: 501756966	NA07/29/202200:00:00

Supplementary Figure S2. Functional categorization by annotation.

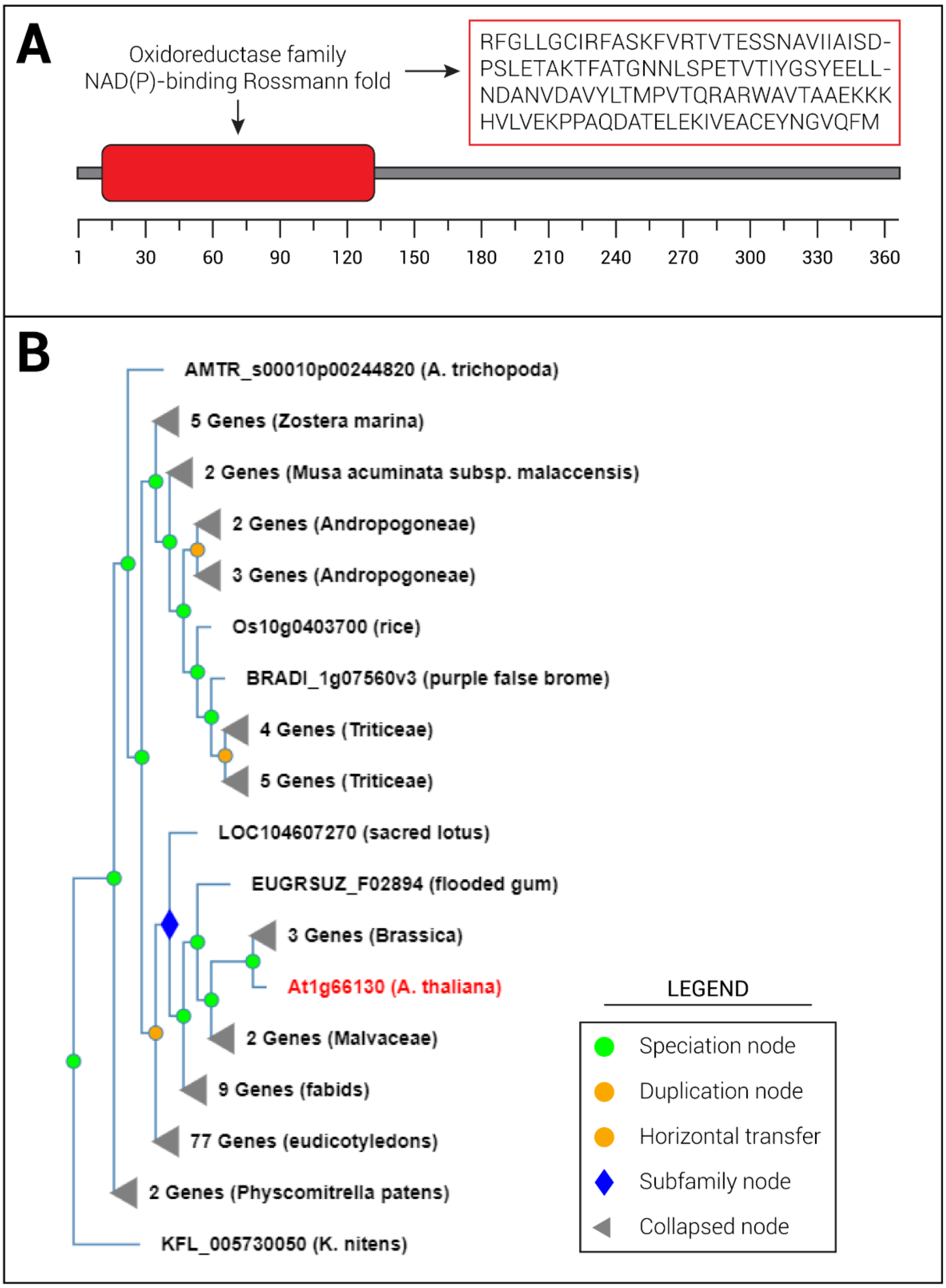

Supplementary Figure S3. Gene structure and phylogenetic analysis.

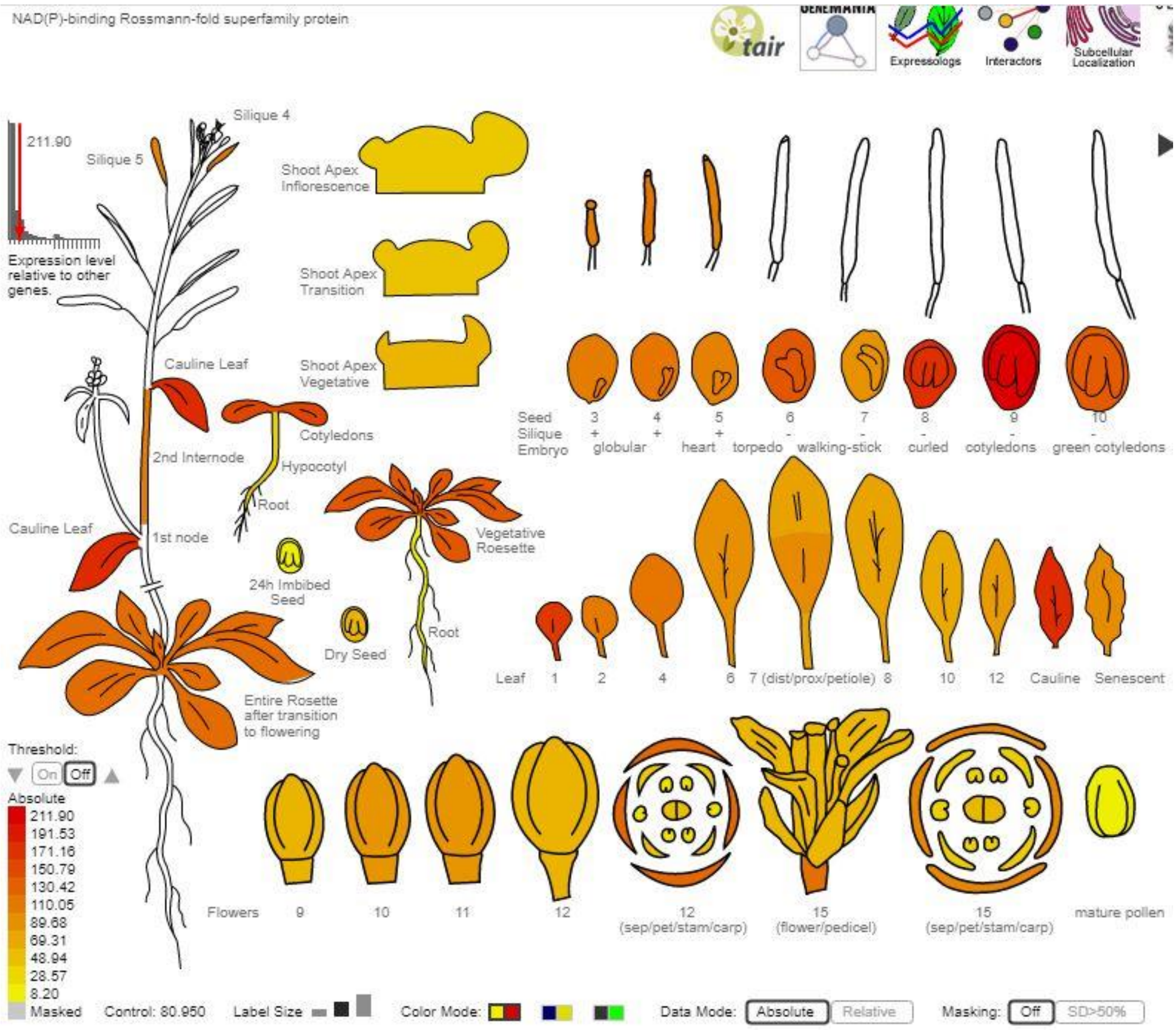

Supplementary Figure S4. Expression of *AtNIGR1* in different tissues of the *Arabidopsis* plant.
